# Analysis of immunotherapeutic response-related signatures in esophageal squamous-cell carcinoma

**DOI:** 10.3389/fimmu.2023.1117658

**Published:** 2023-02-02

**Authors:** Bohao Zheng, Jie Li, Mengdi Zhang, Pengju Zhang, Weiwei Deng, Yang Pu

**Affiliations:** State Key Laboratory of Medical Molecular Biology, Haihe Laboratory of Cell Ecosystem, Department of Physiology, Institute of Basic Medical Sciences and School of Basic Medicine, Chinese Academy of Medical Sciences and Peking Union Medical College, Beijing, China

**Keywords:** esophageal squamous-cell carcinoma (ESCC), immunotherapy, prognosis, tumor microenvironment, immunotherapeutic responsiveness, single-cell RNA sequencing

## Abstract

**Background:**

Esophageal squamous cell carcinoma (ESCC) is one of the most common and lethal malignant diseases. Immunotherapy has been widely studied and has exhibited potential in ESCC treatment. However, there are only a portion of ESCC patients have benefited from immunotherapy. We herein identified immunotherapeutic response-related signatures (IRRS) and evaluated their performance in ESCC prognosis and immunotherapeutic responsiveness.

**Methods:**

We constructed an IRRS using the gene expression data of 274 ESCC patients based on y -30significantly differentially expressed genes, which were compared responders and non-responders from various patient cohorts treated with immunotherapy. Survival analysis was performed in both the GSE53625 and TCGA-ESCC cohorts. We also explored the differences in the tumor microenvironment between the high-IRRS and low-IRRS score groups using single-cell data as a reference. Three immunotherapy cohorts were used to verify the value of the IRRS in predicting immunotherapy response.

**Results:**

Twelve immunotherapy-related genes were selected to construct a signature score and were validated as independent prognostic predictors for patients with ESCC. Patients with high IRRS scores exhibited an immunosuppressive phenotype. Therefore, patients with low IRRS scores may benefit from immunotherapy.

**Conclusions:**

IRRS score is a biomarker for immunotherapy response and prognosis of ESCC.

## Introduction

Esophageal cancer (EC) ranks the 9th most common cancer worldwide and 6th leading cause of cancer deaths globally (1). Esophageal squamous cell carcinoma (ESCC) is the most prevalent EC subtype. In low-income countries, ESCC accounts for over 90% of all EC cases (2–4). Surgical resection is the most primarily and effective treatment for early stage ESCC (5). Chemotherapy (fluoropyrimidine combined with oxaliplatin) is global standard first-line therapy for advanced ESCC (6). Preoperative chemoradiation and targeted therapy are optional strategies for ESCC (7, 8). Nevertheless, these therapy strategies provide only mild survival benefits for advantaged ESCC patients. Most ESCC cases are difficult to diagnose at early stage, with a 5-year survival rate of 20%–30% (9, 10).

Immunotherapy of cancer has achieved significant success in the treatment of various malignancies (11). In recent decades, many trials have been conducted to assess immunotherapies as prospective treatment options for ESCC. Immune checkpoint blockade has been considered as sequential therapy or second-line therapy for advanced ESCC (12). Nivolumab group has been reported to show better median overall survival time (10.9 months) than chemotherapy group (8.4 months) (12). Pembrolizumab achieved an objective response rate of 14.3% in patients with ESCC and had better effective in high *PD‐L1* expression patients (13). However, not all cancer patients respond well to immunotherapy, and it is difficult to identify the subsets of patients who are expected to benefit from immunotherapy. Multiple factors, including remodeling of the tumor microenvironment (TME), immune cell infiltration, and checkpoint expression, may be determinants of the response to immunotherapy (14–16). The interaction between cells in the tumor microenvironment might affect anticancer treatment (17). Recently, single-cell RNA sequencing can distinct cellular populations and provide cell-type-specific gene expression patterns (6). The deconvolution algorithms has been proved to enable estimates cellular populations in bulk sequencing data (18). Integrated single-cell and bulk RNA sequencing analysis enable us to get a further understanding of TME. However, better prognostic tools and biomarkers are required to accurately predict tumor characteristics and immunotherapeutic responsiveness.

In the present study, we established a novel risk model based on immunotherapeutic response-related genes and systematically explored their potential importance as predictive biomarkers for prognosis and immunotherapy response. Our risk signature model can be used to guide individualized ESCC treatment.

## Methods

### Acquisition and processing of datasets

Two ESCC datasets (GSE53625 and GSE160269) were downloaded *via* the Gene Expression Omnibus (GEO) database (https://www.ncbi.nlm.nih.gov/geo/). The GSE53625 dataset comprised 179 ESCC samples and 179 adjacent, matching non-tumor tissues. The single cell RNA (scRNA) sequencing data GSE160269 dataset was composed of 208,659 single cells from 60 ESCC samples.

In addition, 95 ESCC samples were downloaded from The Cancer Genome Atlas (TCGA; https://portal.gdc.cancer.gov). The melanoma immunotherapy cohorts (GSE78220 and GSE91061) were also downloaded from the GEO database. Immunotherapy cohort IMvigor210 was downloaded from the IMvigor210CoreBiologies package (19). Gastric adenocarcinomas(STAD) immunotherapy cohort was downloaded from the Cancer Research Institute (CRI) iAtlas platform(https://isb-cgc.shinyapps.io/iatlas/) (20). The details of these cohorts were summarized in **Supplement Table 2**.

For prognostic analysis, eligible subjects with the standard that available follow-up and prognostic information.

A list of genes, which have significant difference of expression between responders and non-responders in immunotherapy cohorts, was obtained from the TISIDB database (http://cis.hku.hk/TISIDB/) (21). Genes that met the cutoff criteria of false discovery rate (FDR) < 0.05 and |log2-fold change (FC)| > 1 were considered immunotherapeutic response-related genes (IRRGs). A total of 798 genes were included in this study after their intersection with the datasets.

### Construction and validation of the immunotherapeutic response-related signature

IRRGs differentially expressed between paracancerous (n = 179) and cancerous (n = 179) tissues were identified using the R limma package(3.50.3) (22) based on the thresholds of an adjusted p < 0.05 and | log2 (fold change) | > 1. IRRGs significantly associated with overall survival (OS) in ESCC were identified by univariate Cox regression using the survival package (3.2-11) in R. Next, the differentially expressed genes (DEGs) and prognostic genes were investigated using the R ggvenn package (0.1.9) to identify prognostic cellular senescence-related DEGs. The least absolute shrinkage and selection operator (LASSO) Cox regression analysis (23) was performed using the R glmnet package (4.1-4) (24) to construct the risk score model. We performed 1000 substitution samplings in the dataset, which were separated into high- and low-score groups according to the optimal cutoff value of the IRRS as calculated by the cutoff package, and the performance of the IRRS was subsequently evaluated.

### Annotation and functional enrichment analyses

Gene Ontology (GO), Kyoto Encyclopedia of Genes and Genomes (KEGG) and Gene Set Enrichment Analysis (GSEA) analyses were performed using the R cluster Profiler package (4.2.2) (25). DEGs between the high- and low-risk groups were subjected to pathway and functional enrichment analyses. Pathways with P < 0.05, FDR < 0.25 and absolute normalized enrichment score (|NES|) > 1 were considered significant.

### Analysis of scRNA sequencing data from ESCC patients

scRNA-seq data were obtained from the GSE160269 dataset. We profiled the transcriptomes of 208,659 single cells, including 97,631 immune and 111,028 non-immune cells from 60 ESCC samples. The data provided eight main cell populations, namely epithelial cells (N = 44,730), fibroblasts (N = 37,213), endothelial cells (N = 11,267), pericytes (N = 3102), fibroblastic reticular cells (FRC; N = 1,319), T cells (N = 69,278), B cells (N = 22,477), and myeloid cells (N = 19,273) (26). The TME cells were analyzed in this study. The Seurat package (version 4.1.1) (27) was used for quality filtering and downstream analyses. We performed principal component analysis (PCA) with RunPCA, and cell clusters were identified using the “FindNeighbors” and “FindClusters” functions. Cluster-specific marker genes were identified using the “FindAllMarkers” function. The results were visualized on a tSNE plot of the top ten PCs using RunTSNE. Twenty-five immune cells and 16 non-immune stromal cell subtypes were identified in the TME. Pseudotime trajectory analysis was performed using the R monocle package (2.22.0) (28).

### Deconvolution and cell communication analysis

We enumerated the proportions of distinct cell subpopulations in the bulk tissue expression profiles using CIBERSORTx (18) and intercellular communication was evaluated using NicheNet (29). We used a previously described single-cell reference matrix file to create a custom signature matrix. Cell types from the scRNA-seq data were based on prior categorizations.

### Statistical analysis

Data and graphs were all created utilizing the R software (version 4.1.0.). Receiver operating characteristic (ROC) curves were plotted using the R package “timeROC” (0.4). The statistical significance of normally distributed variables was evaluated using unpaired Student’s *t*-tests, and nonnormally distributed variables were estimated using the Wilcoxon rank sum test. Categorical variables between two groups were compared by Fisher’s exact test. Statistical significance was two-sided, and *p* value < 0.05 was considered as the threshold for significance.

## Results

### Identification of differentially expressed immunotherapeutic response-related genes in ESCC

**Figure 1:**
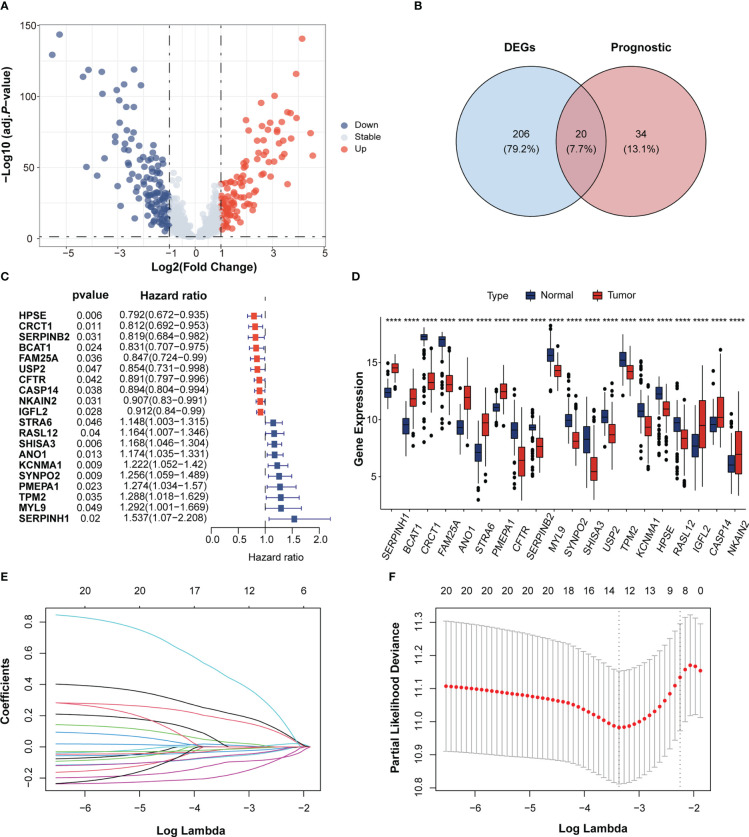
Identification of immunotherapeutic response-related genes in Esophageal squamous-cell carcinoma (ESCC). **(A)** Volcano plot of immunotherapeutic response related genes in the GEO cohort. The blue and red dots indicate down- and upregulated genes. **(B)** Venn diagram of the intersection between the differentially expressed genes and prognostic genes. **(C)** Forrest plot of the univariate Cox regression analysis of 16 overlapping genes. **(D)** The gene expression of 20 overlapping genes between ESCC and normal tissues. Tumor, red; Normal, blue. ****p < 0.0001; ns, no statistical significance; GEO, Gene Expression Omnibus. **(E)** LASSO coefficient profiles of 10 candidate genes. **(F)** Cross-validation for tuning parameter selection in the LASSO regression.

We downloaded a genelist about immunotherapeutic response from the TISIDB database (21) and intersected with the ESCC datasets from GEO and TCGA. 798 genes were obtained. These immunotherapeutic response-related genes were compared between paracancerous and cancerous tissues in the GSE53625 ESCC cohort and 226 DEGs were identified (**Figure 1A**). We used GO and KEGG pathway enrichment analyses to gain insight into the biological process of these DEGs. KEGG analysis revealed that the 226 DEGs were involved in cytokine–cytokine receptor interactions, protein digestion and absorption, complement, and coagulation cascades. GO pathway enrichment analysis demonstrated that the DEGs were enriched in extracellular matrix structural constituents, receptor ligand activity, and signaling receptor activator activity among others (**Figure S1**).

Univariate Cox analysis of the 798 immunotherapeutic response-related genes showed that 54 genes were significantly associated with OS in GSE53625 ESCC cohort. (p < 0.05; **Supplementary Table 1**). Among the 54 genes, 20 genes were overlapped with the 226 DEGs (**Figures 1B, D**). Cox analysis of these 20 overlapping genes showed 10 genes to be protective factors with a Hazard ratio (HR) < 1 and the other 10 genes to be risk factors with an HR > 1 for ESCC prognosis (**Figure 1C**). These results implied that the expressions of these 20 overlapping genes may exert important influence in ESCC progression and prognosis.

### Establishment of the IRRS in ESCC

**Figure 2:**
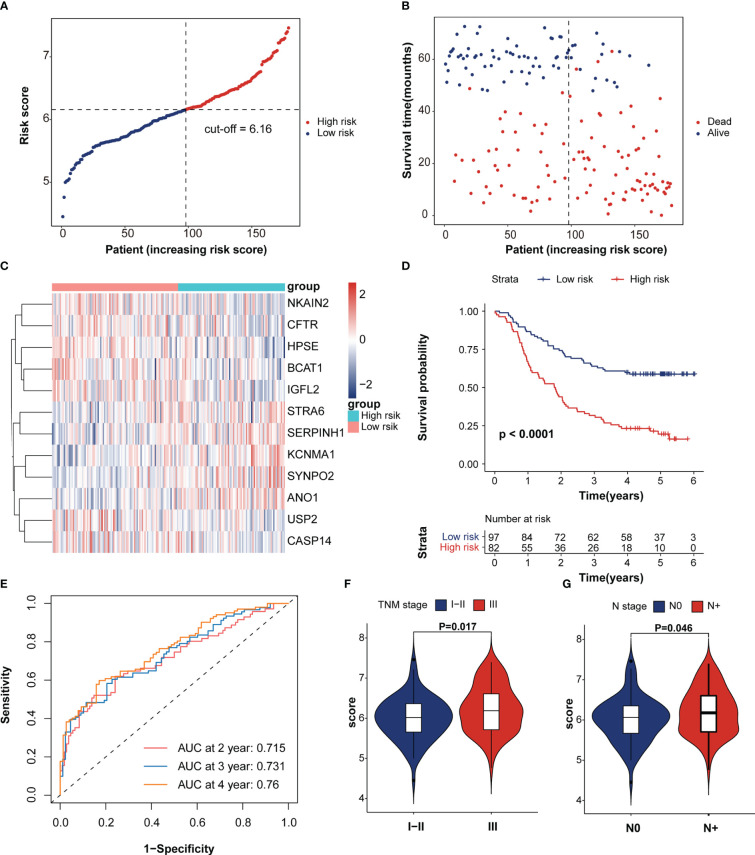
Survival analysis of immunotherapeutic response-related signature. **(A)** The rank of IRRS scores. **(B)** Survival status in the training cohort. **(C)** Heatmap of expression levels of twelve genes in the high- and low-score groups. **(D)** Kaplan–Meier curve of training cohort grouped by IRRS score. **(E)** Time-dependent ROC curve analysis of the prognostic model (2, 3, and 4 years). **(F, G)** The correction between IRRS score and patient’s clinicopathological stage, including T stage and TNM stage.

The LASSO algorithm was used to obtain the coefficients of the 20 genes mentioned above and to construct an immunotherapeutic response-related signature (IRRS) for survival prediction. Based on the optimum λ value, twelve genes were selected to be the IRRS, indicating the prognostic signature (**Figures 1E, F**). The risk score of each patient was calculated based on the formula: risk score = (0.02884450*expression value of *STAR6*) + (-0.09624035*expression value of *HPSE*) + (0.21499692*expression value of *KCNMA1*) + (0.17381030*expression value of *ANO1*) + (0.44276994*expression value of *SERPINH1*) + (-0.18003164*expression value of *BCAT1*) + (-0.01284725*expression value of *USP2*) + (-0.02837151*expression value of *NKAIN2*) + (-0.06109935*expression value of *IGFL2*) + (-0.05416382*expression value of *CASP14*) + (-0.01472970*expression value of *CFTR*) + (0.01164075*expression value of *SYNPO2*). Patients in the training cohorts were classified into low- and high-risk groups based on the cut-off value of the risk score (**Figures 2A–C**). Kaplan–Meier curve demonstrated that the prognosis of ESCC patients in the high-risk group was significantly poorer than that in the low-risk group (*p* < 0.0001; **Figure 2D**). We then performed Time-dependent ROC analysis and found the areas under the curve (AUCs) of the IRRS model for the 2-, 3-, and 4-year OS were 0.715, 0.731, and 0.76, respectively (**Figure 2E**). The correlation between the IRRS and patients’ clinicopathological characteristics, including age, sex, drinking and smoking status, N stage, T stage and TNM stage (**Figure S2**) was demonstrated. Furthermore, patients with late-stage ESCC had higher IRRS scores (**Figure 2F, G**). However, there was no significant correlation between IRRS score and other clinicopathological factors.

To verify the prognostic value of the IRRS, the TCGA-ESCC cohort was enrolled using the same method applied to the training dataset. The analysis revealed that patients in the high-risk score group have a poorer prognosis (*p* = 0.044; **Figure S3A**). The area under the time-dependent ROC curve (AUC) values in the TCGA-ESCC cohort were 0.823, 0.811, and 0.844 at 2, 3, and 4 years, respectively (**Figure S3B**).

**Figure 3:**
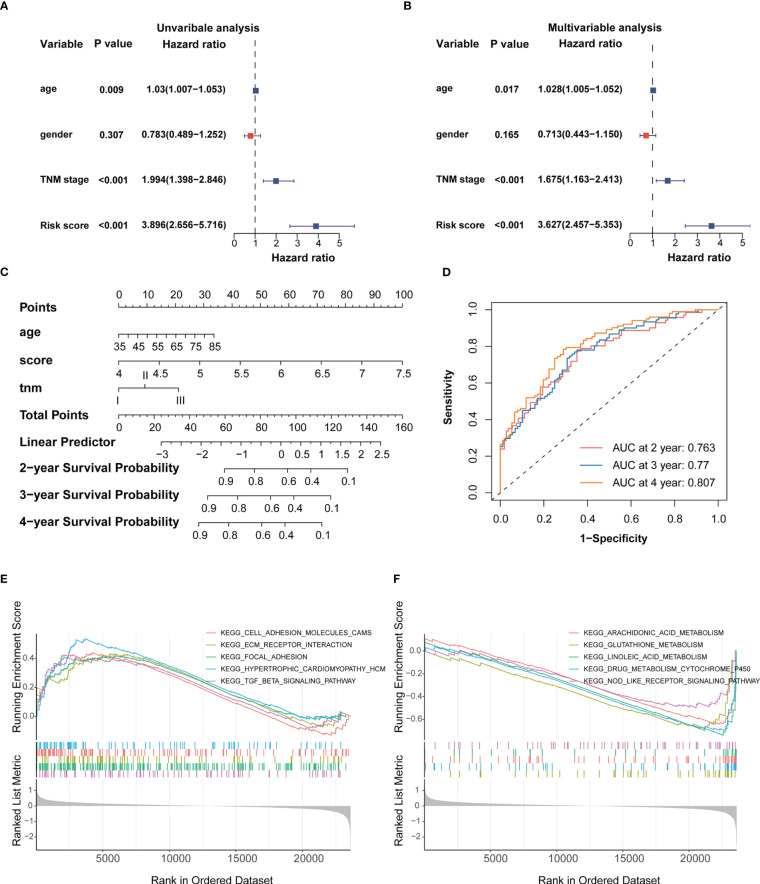
Establishment of the IRRS score-based nomogram **(A, B)** Forest plot of univariable and multivariable Cox regression analysis of IRRS and overall survival in the training cohort. **(C)** Nomogram to predict the probability of OS in 2, 3 and 4 years for ESCC. **(D)** AUC values of ROC predicted 2, 3 and 4 year OS rates of Nomogram. **(E)** GSEA analysis of the high IRRS score group. **(F)** GSEA analysis of the low IRRS score group.

Univariate and multivariate Cox analyses indicated that age, TNM stage and IRRS score were independent prognosticfactors for the ESCC patients (**Figure 3A, B**). We then constructed a predictive nomogram to improve the prognosis capacity of the IRRS score model and to provide a visualization and quantitative method for predicting 2-, 3-, and 4-year OS (**Figure 3C**). The concordance index (C-index) of this model was 0.704, and the AUCs of the 2-, 3-, and 4-year OS for the nomogram were 0.763, 0.77, and 0.807, respectively (**Figure 3D**), which were superior to the prognostic efficacy of the IRRS score alone (0.715.0.731 and 0.76, respectively). We plotted calibration curves to evaluate the performance of the nomogram. The results showed the model’s predictions curve were close to the ideal curve (**Figures S4A–C**). These results suggested that the nomogram model has a great prediction power for ESCC patients.

Esophageal squamous cell carcinoma is a disease with highly somatic alterations. We used validation cohort TCGA-ESCC cohort to detected mutation. The top 30 mutated genes in the high and low SIRGs score groups are shown in **Figures S5A, B**. ESCC driver TP53 was high in high risk group compared with low risk group (**Figures S5C, D**) (30). The IRRS score was no difference between high- and low- risk group (**Figure S5E**).

To provide the mechanistic explanation for the predictive significance of IRRS and describe the possible mechanisms underlying the predictive role of the signature, we performed differential gene expression analyses on the training cohort. DEGs between the two groups were identified by applying the screening threshold of FDR < 0.05 and |log2FC| ≥ 1, GO and KEGG pathway analyses were performed based on these DEGs. The results showed that immune responses, such as interleukin-1 receptor binding and the IL-17 signaling pathway, were enriched in these DEGs (**Figure S4G**). In addition, GSEA analysis revealed that focal adhesion, ECM receptor interaction, and TGF-β signaling were significantly enriched in the high IRRS score group (**Figure 3E**), whereas glutathione and linoleic acid metabolism and Nod-like receptor signaling were enriched in the low IRRS score group (**Figure 3F**). Taken together, these results indicated the high risk group may possess immunosuppressive TME.

### Identification of the association between the IRRS and the tumor microenvironment using scRNA sequencing data

**Figure 4:**
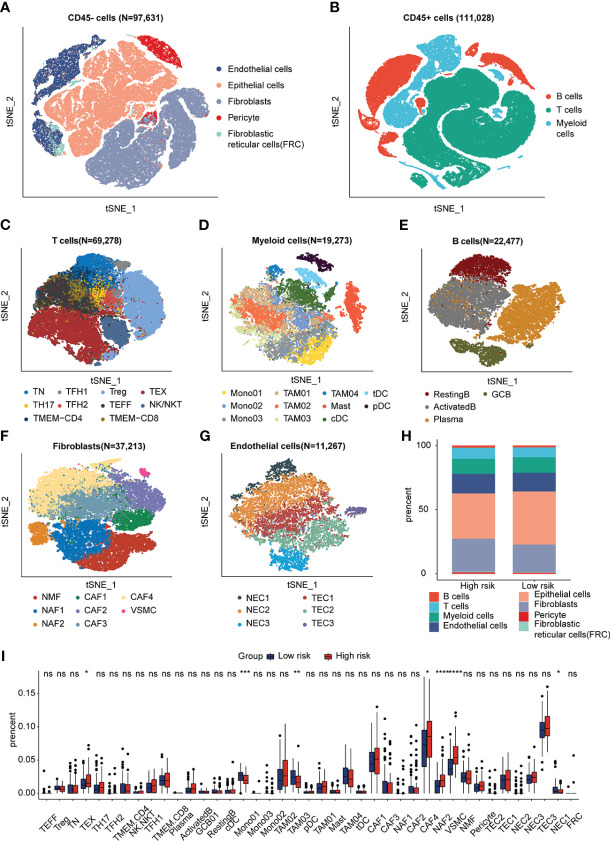
Visualization plots of scRNA-seq data. **(A, B)** tSNE plot of 208,659 cells from ESCC. **(C–G)** tSNE plots of T cells, Myeloid cells, B cells, Fibroblasts and Endothelial cells colored by cell type. **(H)** Fractions of each cell type in low- and high-SIRGs score group. **(I)** The different TME cells in low- and high-IRRS score groups. *p<0.05, **p<0.01, ***p<0.001, ns, not significant.

scRNA sequencing data was analyzed to further explore the synergistic effect of the IRRS and the TME cells in ESCC. We used the previously profiled transcriptomes of ~208,659 single cells with eight main cell populations (**Figures 4A, B**), among which fibroblasts, endothelial cells, T cells, B cells, pericytes and myeloid cells were analyzed. Next, 40 cell clusters, including T helper 17 (TH17) cells, follicular helper T (TFH1/2) cells, naïve T (TN) cells, regulatory T (Treg) cells, memory T (TMEM-CD4/CD8) cells, effector T (TEFF) cells, exhausted T (TEX) cells, monocytes (Mono01-03), tumor-associated macrophages (TAM01-04),dendritic cells (tDC, pDC, cDC), mast cells (Mast), resting B cells, activated B cells, germinal center B cells (GCB), plasma cells, normal mucosa fibroblasts (NMF), normal activated fibroblasts (NAF1/2), cancer-associated fibroblasts (CAF1-4), vascular smooth muscle cells (VSMC), normal endothelial cells (NEC1-3) and tumor endothelial cell (TEC1-3) were identified by t-SNE analysis (**Figures 4C–G**; **Figures S6A–E**). We then used CIBERSORTx to infer cell-type abundance from bulk RNA-seq data using scRNA data, and estimated the proportions of the 42 cell types in the training dataset. The proportions of TME cell types between the high- and low-risk groups was compared using the Kruskal–Wallis test. The profiles of the high- and low-risk groups are shown in **Figure 4H** and **Figure S6F**. The abundance of TEX, NAF2, CAF4, and VSMC increased, and the abundance of Mono1, TAM03, and NEC2 decreased in the high-risk group (*p* < 0.05; **Figure 4I**). The cell proportions between low and high risk groups demonstrated significantly distinct TME patterns.

**Figure 5:**
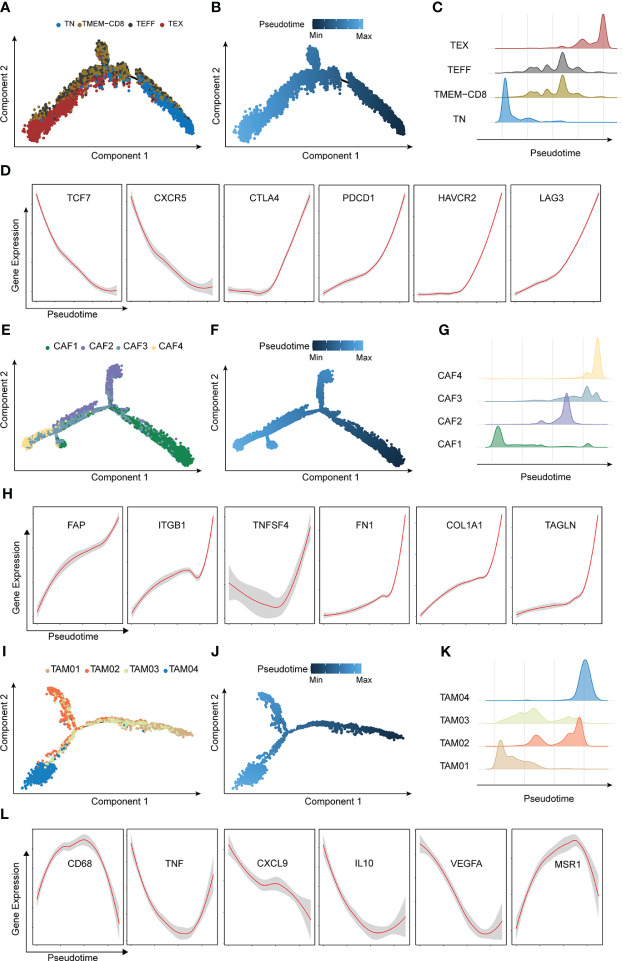
Characterization of major trajectories. **(A)** Pseudotime trajectory of CD8+T cells colored by cell types. **(B)** Pseudotime trajectory of CD8+T cells colored by pseudo-time. **(C)** Distribution of CD8+T cells. **(D)** Expression of CD8+T marker genes across pseudotime. **(E)** Pseudotime trajectory of cancer-associated fibroblasts colored by cell types. **(F)** Pseudotime trajectory of cancer-associated fibroblasts colored by pseudotime. **(G)** Distribution of cancer-associated fibroblasts. **(H)** Expression of tumor-associated macrophages marker genes across pseudotime. **(I)** Pseudotime trajectory of CD8+T cells colored by cell types. **(J)** Pseudotime trajectory of tumor-associated macrophages colored by pseudotime. **(K)** Distribution of tumor-associated macrophages. **(L)** Expression of tumor-associated macrophages marker genes across pseudotime.

The roles of these cells in the different groups were investigated. The trajectories of CD8^+^ T cells, CAFs, and TAMs were calculated using the Monocle method. The trajectory of CD8^+^ T cells ranged from TN to TEX, TEFF, and TMEM, with CD8 being an intermediate cluster (**Figures 5A–C**). As expected, *TCF7* and *CXCR5* were downregulated during the pseudotime, but immune checkpoint genes such as *PDCD1*, *CTLA4*, *LAG3*, and *HAVCR2* increased along the pseudotime axis (**Figure 5D**). The trajectory of CAFs was from CAF1 to CAF4 (**Figures 5E, F**), and markers of CAFs with immunosuppressive functions (31)–*FAP*, *ITGB1*, and *TNFSF4*–were upregulated along the pseudotime axis. ECM markers such as *FN1*, *COL1A1*, and *TAGLN* were also gradually enriched along the trajectory. (**Figure 5G**). The pseudo-ordering of tumor-associated macrophages is organized into two main branches (**Figures 5H–K**). The division of the TAMs is complex. TAMs display remarkable plasticity and are regulated by their local microenvironment (32). M1 macrophage markers (*CD68*, *TNF*, and *CXCL9*) and M2 macrophages markers (*IL10*, *VEGFA*, *MSR1*) were expressed in both intermediate clusters of TAM03 (**Figure 5L**). We considered TAM03 to be in a state of constant transition between the different TAM types.

These results implied that high-level IRRS may exhibit an immunosuppressive phenotype mediated by the high abundance of exhausted T cells and CAFs.

### NicheNet revealed intercellular communication

**Figure 6:**
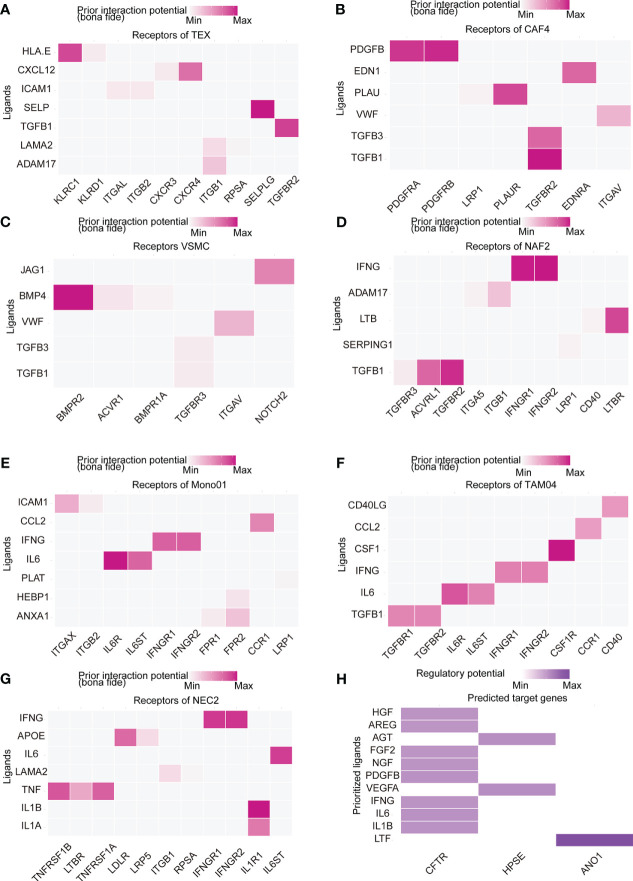
Cell–cell communications result. **(A–G)** Heatmap visualizing NicheNet analysis of receptors of prioritized ligands expressed by **(A)** exhausted T (TEX) cells, **(B)** cancer-associated fibroblasts (CAF4) cells, **(C)** vascular smooth muscle cells (VSMC), **(D)** normal activated fibroblasts (NAF2), **(E)** tumor-associated macrophages (TAM03), **(F)** monocytes (Mono01), and **(G)** normal endothelial cells (NEC2). **(H)** NicheNet analysis of ligand-receptor interactions of IRRS.

**Figure 7:**
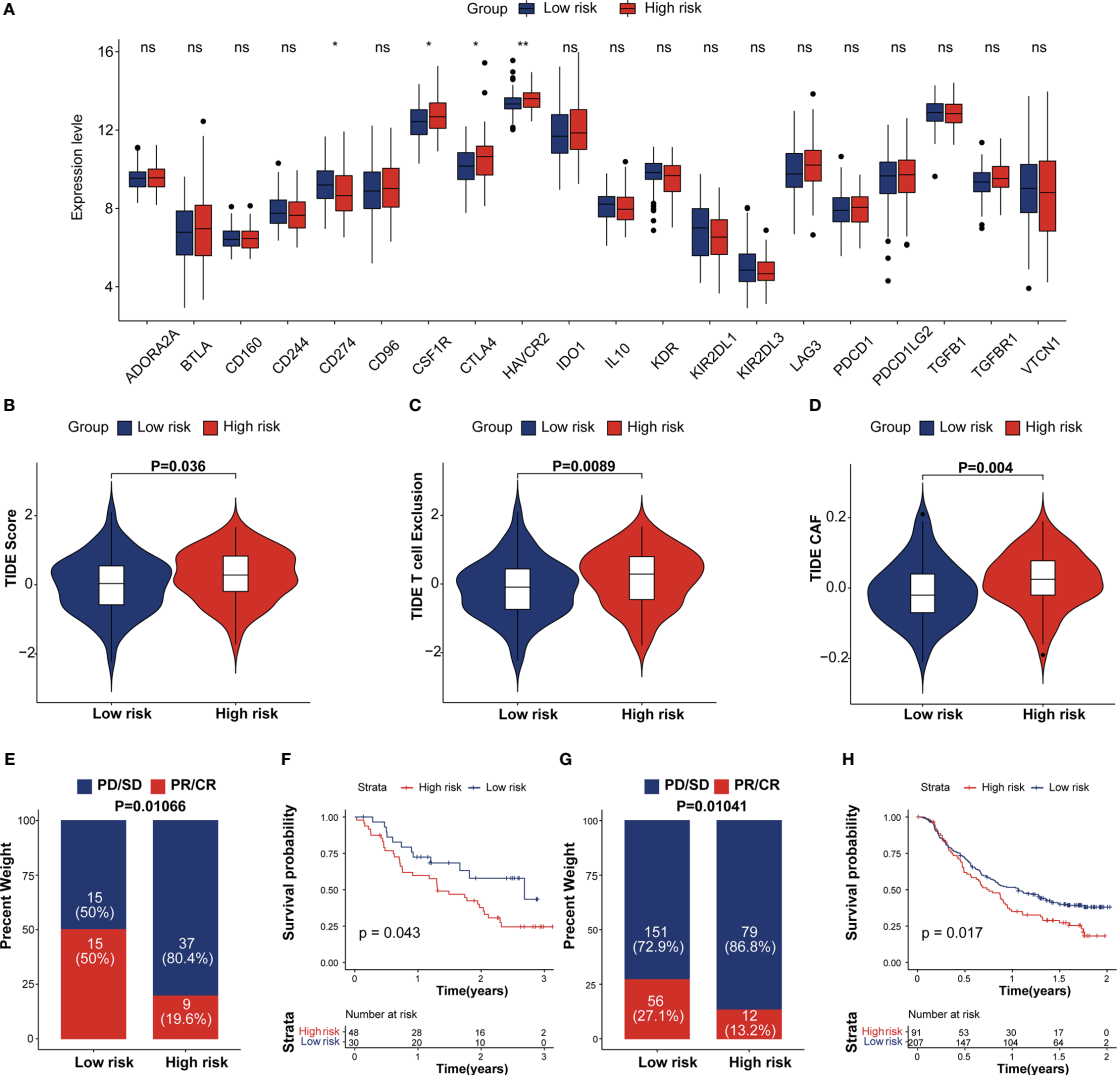
Estimation and validation of IRRS in immunotherapeutic. **(A)** The expression levels of 20 immune checkpoint genes in different IRRS score groups. *p < 0.05; **p < 0.01; ns, no statistical significance; **(B–D)** The distribution of TIDE scores, T cell excel in the high-risk and low-risk groups. **(E)** The distribution of IRRS scores between two immunotherapy response groups in the melanoma cohort. **(F)** Kaplan–Meier curves for high and low IRRS score patient groups in the melanoma cohort. **(G)** The distribution of IRRS scores between the two immunotherapy response groups in the IMvigor210 cohort. **(H)** Kaplan–Meier curves for high and low IRRS score patient groups in the IMvigor210 cohort.

To further identify the TME interaction between IRRS subtypes, intercellular communication was inferred using NicheNet analysis, a computational method that models cell−cell communication by using prior knowledge to prioritize ligand–receptor pairs. We inferred that the modes of intercellular communication differed by risk group. TGFB1-related ligand−receptor pairs were predicted to be increased in the high-risk group (**Figures 6A–D**), and IFNG-related ligand−receptor pair receptors were predicted to be increased in the low-risk group (**Figures 6E–G**). *CXCL12*-*CXCR4* and *HLA*-*E*-*KLRC1* were predicted in TEX cells compared to other cell types (**Figure 6A**); *PDGFRB*-*PDGFRA* expression was predicted in CAF4 cells (**Figure 7B**); *BMP4*-*BMPR2* was predicted in VSMCs (**Figure 6C**); and *CD40LG*-*CD40* was predicted in TAM03 cells (**Figure 6F**) and increased the antitumor immune response (33, 34). The complex crosstalk in the TME indicates different immunosuppressive levels between high- and low-risk groups. We used only 12 IRRS genes for NicheNet analysis in differentially expressed cell types to predict the target ligands of IRRS (**Figure 6H**), and these ligand–receptor interactions may be applied as a guide for immunotherapy.

### Predictive potential of the IRRS for immunotherapy response

Recent studies showed that the immune checkpoint associated-genes can modulate immune infiltration (35). We compared the expression of 20 inhibitory immune checkpoint molecules between high and low IRRS group. (**Figure 7A**). CTLA4 and HAVCR2, which are exhausted T-cell markers, were found expressed at high levels in the high IRRS group whereas PD-L1 tend to be expressed at high levels in the low IRRS group. We then analyzed the correlation between the IRRS and Tumor Immune Dysfunction and Exclusion (TIDE), which are recognized as immunotherapy predictors (36, 37). As expected, the differences observed in the TIDE analysis were similar to those observed in the scRNA analysis. We found that patients in the high-risk group tended to achieve higher T-cell exclusion, CAF and TIDE scores. (**Figures 7B–D**). More importantly, we performed Kaplan–Meier survival analysis to investigate the predictive role of immunotherapy on OS using the immunotherapy cohort, including the IMvigor210, STAD and melanoma cohorts (GSE78220 and GSE91061). In the IMvigor210 cohort, patients in the complete response (CR) and partial response (PR) groups tended to achieve lower IRRS scores (**Figure 7E**). As depicted in **Figure 7F**, low-risk patients had prolonged OS compared with high-risk patients. A similar result was observed in the melanoma and STAD cohorts (**Figures 7G, H**; **Figure S7**). These results proposed that patients with low IRRS scores may benefit from immunotherapy.

## Discussion

ESCC is one of the most common esophageal cancer subtypes in Asian populations, only a subset of ESCC patients can benefit from immunotherapy. It is important to identify the population of patients expected to respond to immunotherapy and their specific TME. In our study, We established a survival prediction model -IRRS by the gene expression of twelve senescence features in the GEO dataset. The IRRS was validated using the TCGA cohort and displayed robust predictive capability. We also used scRNA-seq as a reference to characterize cell types within TME. Two external immunotherapy cohorts were chosen to verify the efficacy of the IRRS score in predicting immunotherapy response. The results provide new insights for deep understanding of the mechanism for prognosis and immunotherapy response associated with the TME, which may benefit ESCC patients’ precision care.

Twelve immunotherapeutic response-related genes (*STRA6*, *HPSE*, *KCNMA1*, *ANO1*, *SERPINH1*, *BCAT1*, *USP2*, *NKAIN2*, *IGF12*, *CASP12*, *CFTR*, and *SYNPO2*) were herein selected to establish the IRRScore prognostic model. These genes have been reported to associated with tumor progression and immune suppressive TME in ESCC and other cancers. Specifically, *HPSE*, *ANO1*, and *SERPINH1* have been shown to be prognostic indicators of ESCC (38–41). *STRA6*, *IGFL2*, *USP2* and *CASP14* are associated with the prognosis of other cancers, including hepatocellular carcinoma, bladder cancer, gastric cancer and clear cell renal cell carcinoma (42–48).The overexpression of CFTR suppressed the proliferation and migration/invasion of ESCC cells and was associated with a good patient prognosis (49). SERPINH1 and BCART1 are associated with various immune checkpoint genes in some cancers (41, 50, 51). The absence of NK cell-heparanase impaired the effect of immune checkpoint blockade immunotherapy (39). KCNMA1, a large potassium (BK) ion channel, is a possible target for cancer immunotherapy (52). SYNPO2 and CFRT expression play an important role predicting the efficacy of immune checkpoint inhibitor therapy (53, 54). Based on the previous studies, we believe that the IRRS has the potential to reflect ESCC prognosis based on the alterations of immune landscape.

scRNA-seq enables a comprehensive investigation of cell diversity in heterogeneous ESCC tissue samples. We deconvoluted the bulk ESCC RNA-seq data using ESCC scRNA-seq data to estimate cell-type proportions to avoid errors from changes in expression patterns. TEX, CAF4, NAF2 and VSMCs proportions were significantly higher in ESCC patients in the high risk group. TEX is characterized by elevated expression of inhibitory receptors (55).Trajectory analysis revealed that CAF1 was transformed into CAF4. The markers of CAFs, constituting an immunosuppressive environment, increased along the pseudotime (31). The higher abundance of TEX and CAF4 suggests that patients with higher IRRS scores may have an immunosuppressive TME. TAMs exhibit a high degree of plasticity, and TAM03 expressed both M1 and M2 macrophage markers, suggesting that it is sensitive to the internal environment.

The interactions between immune and non-immune stromal cells may promote an immunosuppressive ESCC TME. TGF-β-related ligand−receptor pairs were predicted in high-risk group cell types, with GSEA also showing upregulated TGF-β signaling; this was reported to be an antitumor immunity restraining factor (19, 56, 57). On the other hand, IFN-γ-related ligand−receptor pairs, which have been reported to be necessary for clinical benefit during immunotherapy (58), were predicted in the low-risk group cell types. The expression of exhausted T-cell markers, *HAVCR2* and *CTLA-4*, were also higher in ESCC specimens with high IRRS scores. Interestingly, *PD-L1* expression increased in ESCC specimens with low IRRS scores. Participants with high *PD‐L1* expression had a higher OS rate than those with low *PD‐L1* expression (59). In our research, low IRRS score group had higher TIDE score and *PD-L1* expression, suggesting that they were more able to benefit from PD-1/PD-L1 inhibitors treatment (36, 37, 59).

We used two external immunotherapy cohorts to further verify the efficacy of IRRS score. In this result, low-risk patients had a prolonged OS time compared to high-risk patients in the three immunotherapy cohorts (IMgovir210, melanoma and STAD cohorts). There is evidence indicates that exhausted T-cells leading to poor responsiveness to treatment with immune checkpoint blockade (13). The complex interaction that occurs between TME immune checkpoint genes and IRRS may provide new insights into patients who will benefit from treatment with immune checkpoint inhibitor The immunotherapeutic response-related signature can be used not only as a prognostic tool but also as a guide for individualized immunotherapy. In our study, *ANO1*, *HPSE* and *CFTR* were predicted to participate in TME crosstalk. Thus, these genes may serve as more accurate biomarkers.

Our study still has some limitations. First, the twelve-gene signature was developed and validated based on a public database; thus, more experimental data are needed. Second, there are 58.1% and 55.8% patients have been treated with chemotherapy or radiotherapy in GSE53625 and TCGA-ESCC cohorts. These proportions are similar to patients with prior treatment in immunotherapy cohorts which has been used as validation cohorts, such as 51.5% in GSE91061, 42.8% in GSE78220 and 78.1% in IMvigor210 cohorts. Since we are establishing a model to predict the survival and immunotherapeutic effect of ESCC patients, whose gene expression status can reflect the gene expression of patients before immunotherapy. The current obtainable ESCC and immunotherapy cohort are very limited. So, we can’t find a cohort which had patients without prior treatment. We will use more accurate cohorts for validation in the future when these dataset is obtainable. Third, the number of immunotherapy cohorts with high-throughput sequencing is very limited. We need to verify the applicability of our research results in patients with ESCC receiving immunotherapy. Moreover, the molecular functions and mechanisms of prognostic IRRS should be further elucidated using basic experiments.

## Data availability statement

The datasets used in this study can be acquired here: https://www.ncbi.nlm.nih.gov/geo/ and https://portal.gdc.cancer.gov/. The accession numbers are included in the article.

## Author contributions

YP and BZ conceived and designed the study. BZ, MZ, JL, and WD analyzed the bioinformatics data. BZ, PZ and YP wrote the manuscript. All authors contributed to the article and approved the submitted version.
