# Supplementary material for: Analysis of immunotherapeutic response-related signatures in esophageal squamous-cell carcinoma
Source: Front Immunol. 2023 Feb 2;14:1117658. doi: 10.3389/fimmu.2023.1117658 (PMC9933905; doi:10.3389/fimmu.2023.1117658)
Supplement: Supplementary file 1 [file DataSheet_1.docx]

Supplementary Material

**Supplementary Figure 1. (A–C)** GO enrichment analysis. **(D)** KEGG pathway enrichment analysis.

**Supplementary Figure 2. (A)** Rank of IRRS scores in the TCGA cohort. **(B)**Survival status in the TCGA cohort. **(C)** Heatmap of expression levels of twelve genes in the high- and low-score groups. **(D)** Kaplan–Meier curve of TCGA cohort grouped by IRRS score. **(E)** Time-dependent ROC curve analysis of the prognostic model (2, 3, and 4 years) in the TCGA cohort.

**Supplementary Figure 3.** Correlation between IRRS score and patient’s clinicopathological parameters, including age, sex, smoking, drinking, and T stage.

**Supplementary Figure 4. (A-C)** Calibration plot of the nomogram to predict the probability of OS at 2, 3 and 4 years. **(D-E)** Go enrichment analysis. **(F)** KEGG pathway enrichment analysis

**Supplementary Figure 5.(A)**Mutational landscape of ESCC patients in high risk group. **(B)**Mutational landscape of ESCC patients in low risk group. **(C)**The summary of mutation in high risk groups. **(D)** The summary of mutation in low risk groups. **(E)** The distribution of TMB in low- and high-risk group.

**Supplementary Figure 6. (A-E)** Dotplot showing expression status of selected markers in each cell cluster.**(F)** The major cell proportions between high- and low-risk groups.

**Supplementary Figure 7. (A)** The distribution of IRRS scores between two immunotherapy response groups in the STAD cohort. **(B)** Kaplan–Meier curves for high and low IRRS score patient groups in the STAD cohort.
