# Supplementary figures and images for: Analysis of immunotherapeutic response-related signatures in esophageal squamous-cell carcinoma
Source: Front Immunol. 2023 Feb 2;14:1117658. doi: 10.3389/fimmu.2023.1117658 (PMC9933905; doi:10.3389/fimmu.2023.1117658)

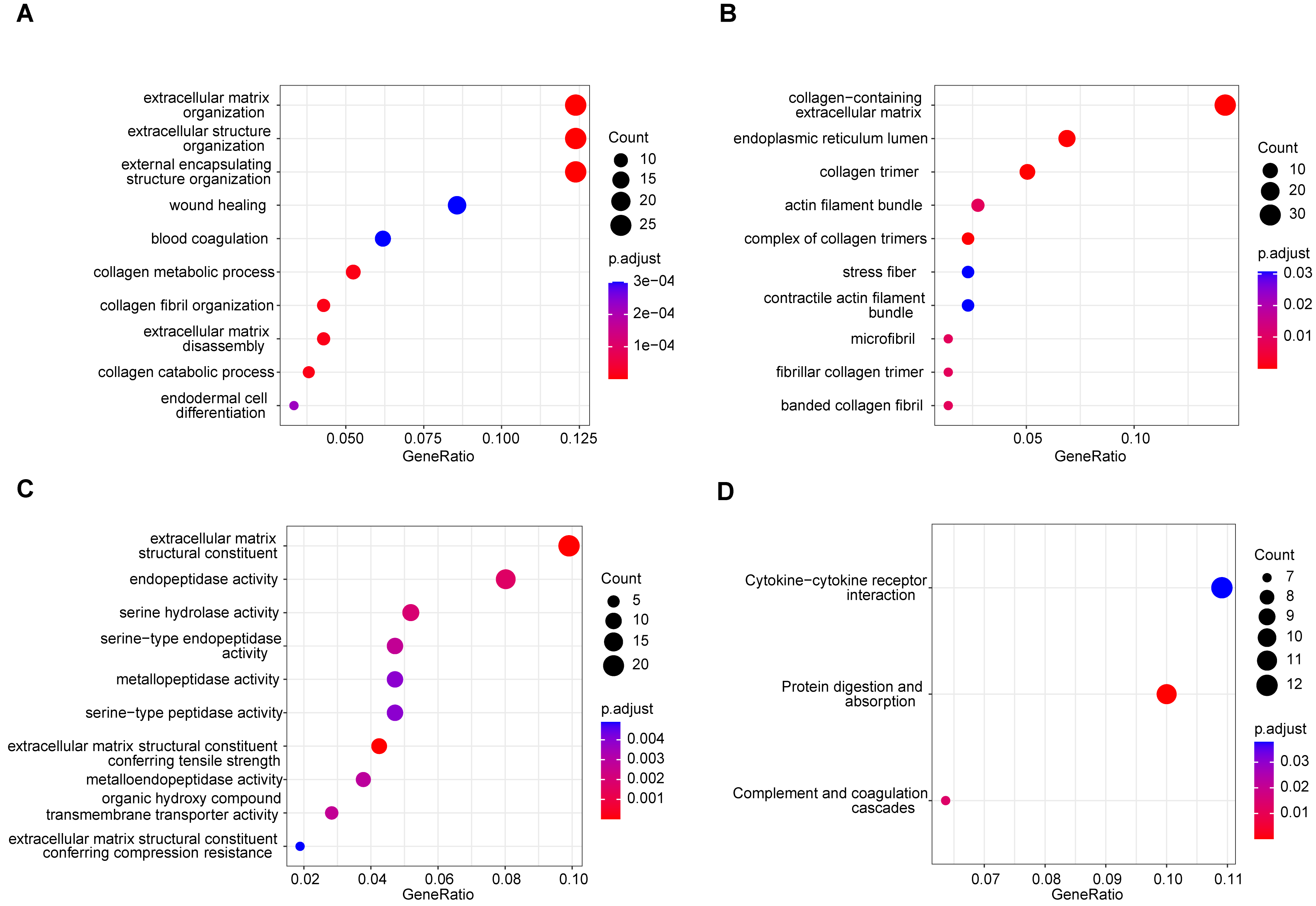

Supplement: Supplementary file 2 [file Image_1.tif]

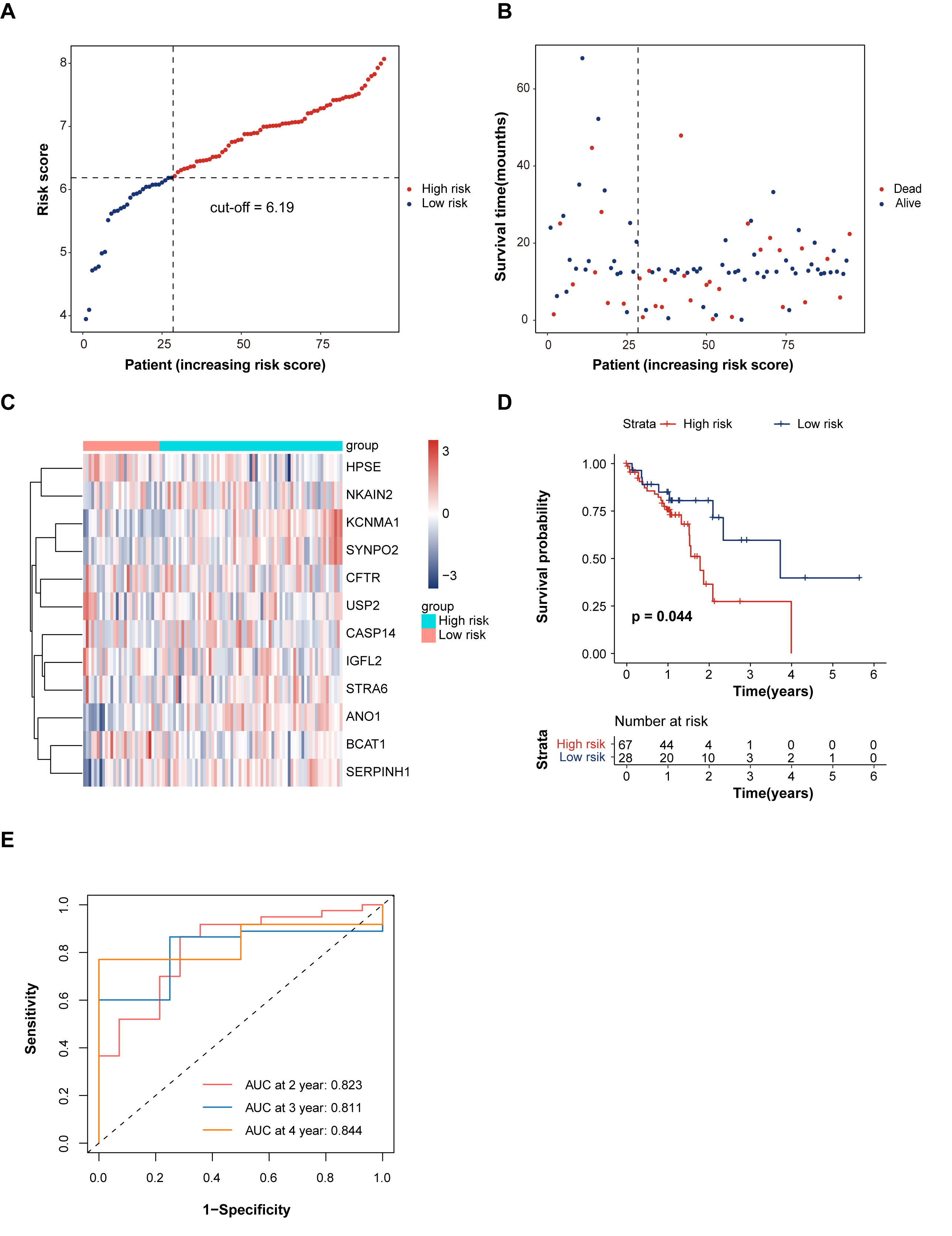

Supplement: Supplementary file 3 [file Image_2.tif]

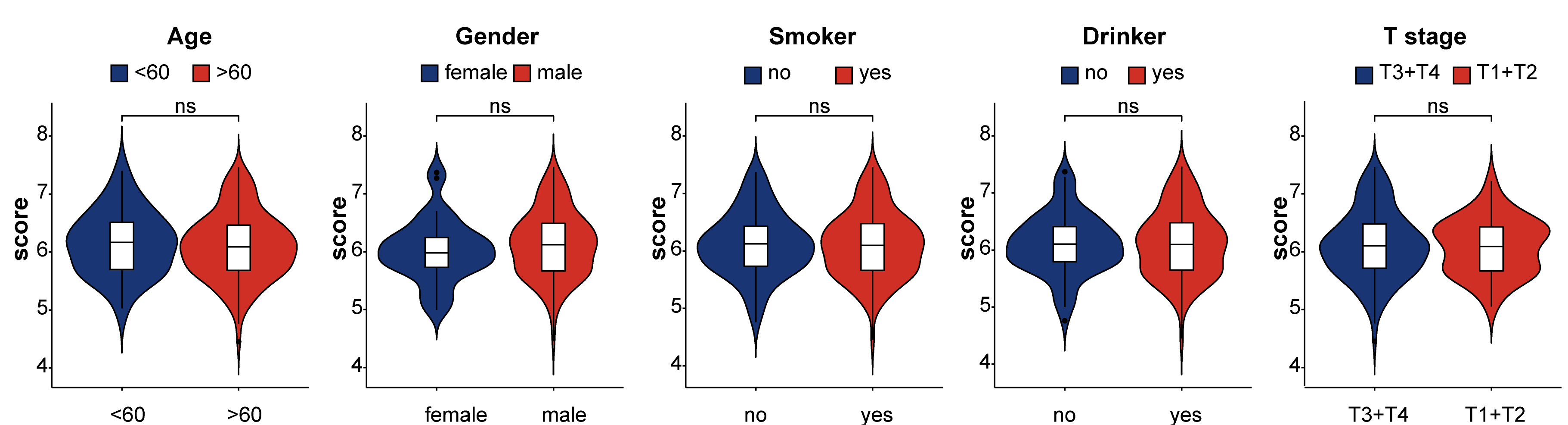

Supplement: Supplementary file 4 [file Image_3.tif]

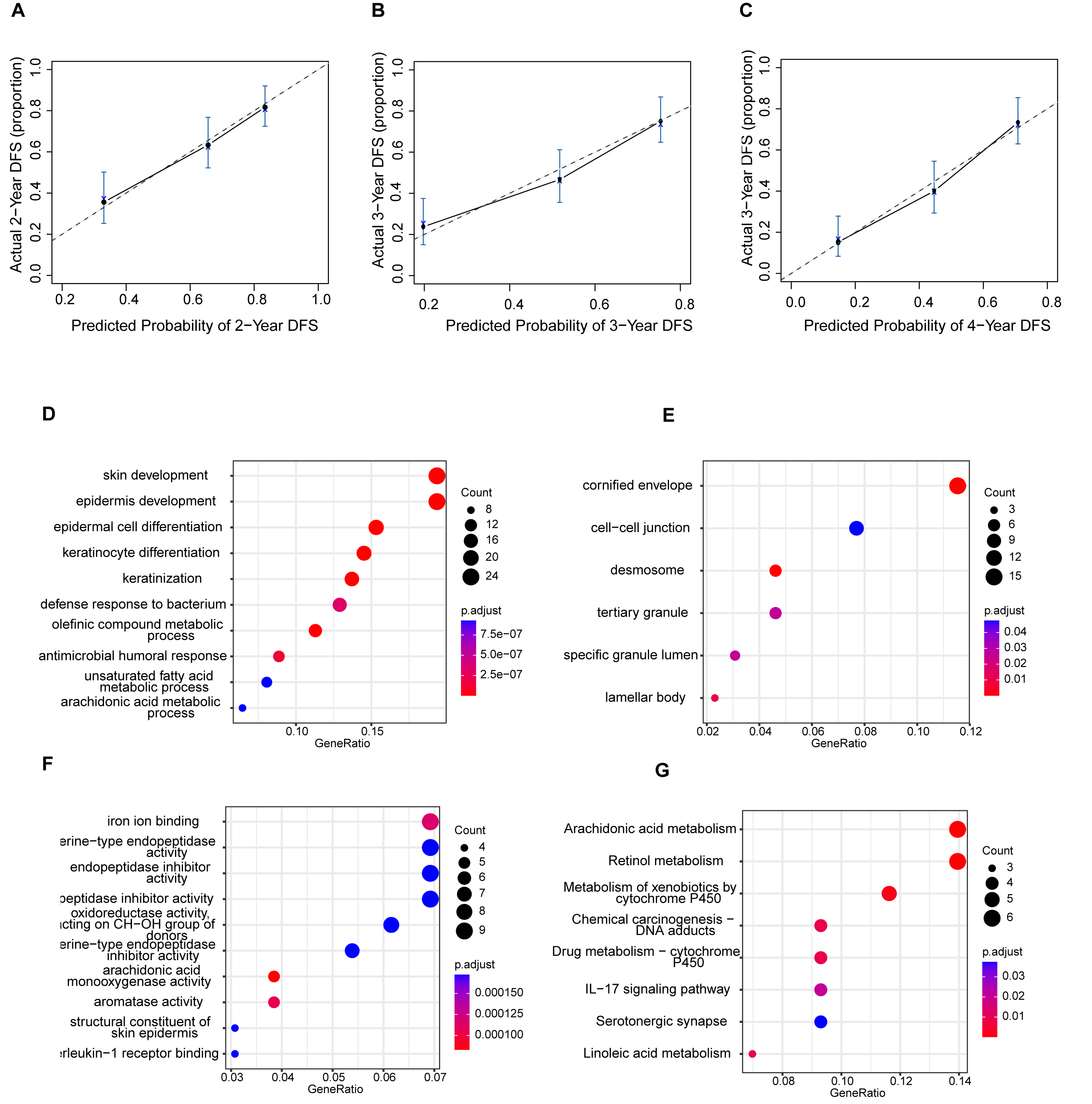

Supplement: Supplementary file 5 [file Image_4.tif]

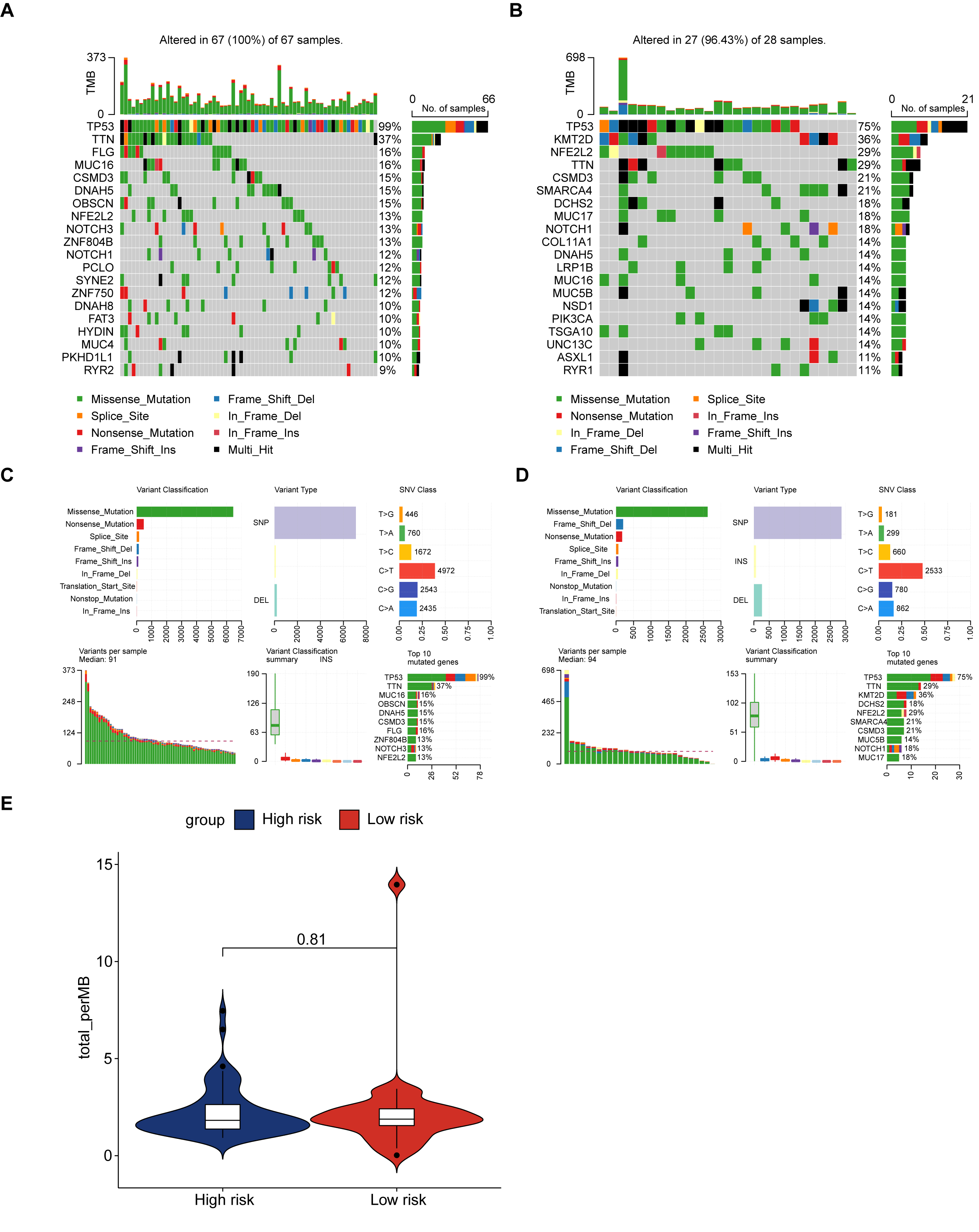

Supplement: Supplementary file 6 [file Image_5.tif]

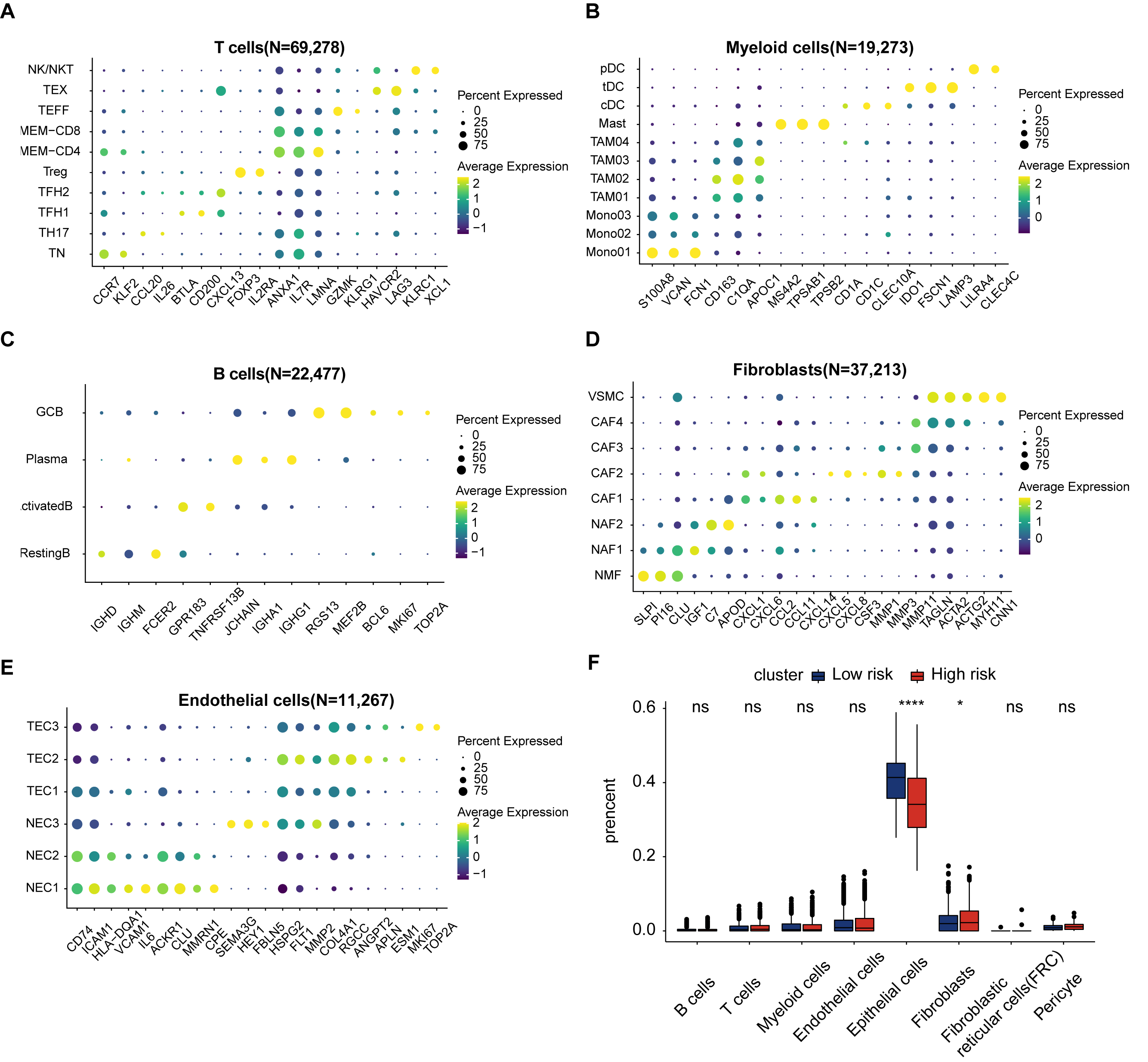

Supplement: Supplementary file 7 [file Image_6.tif]

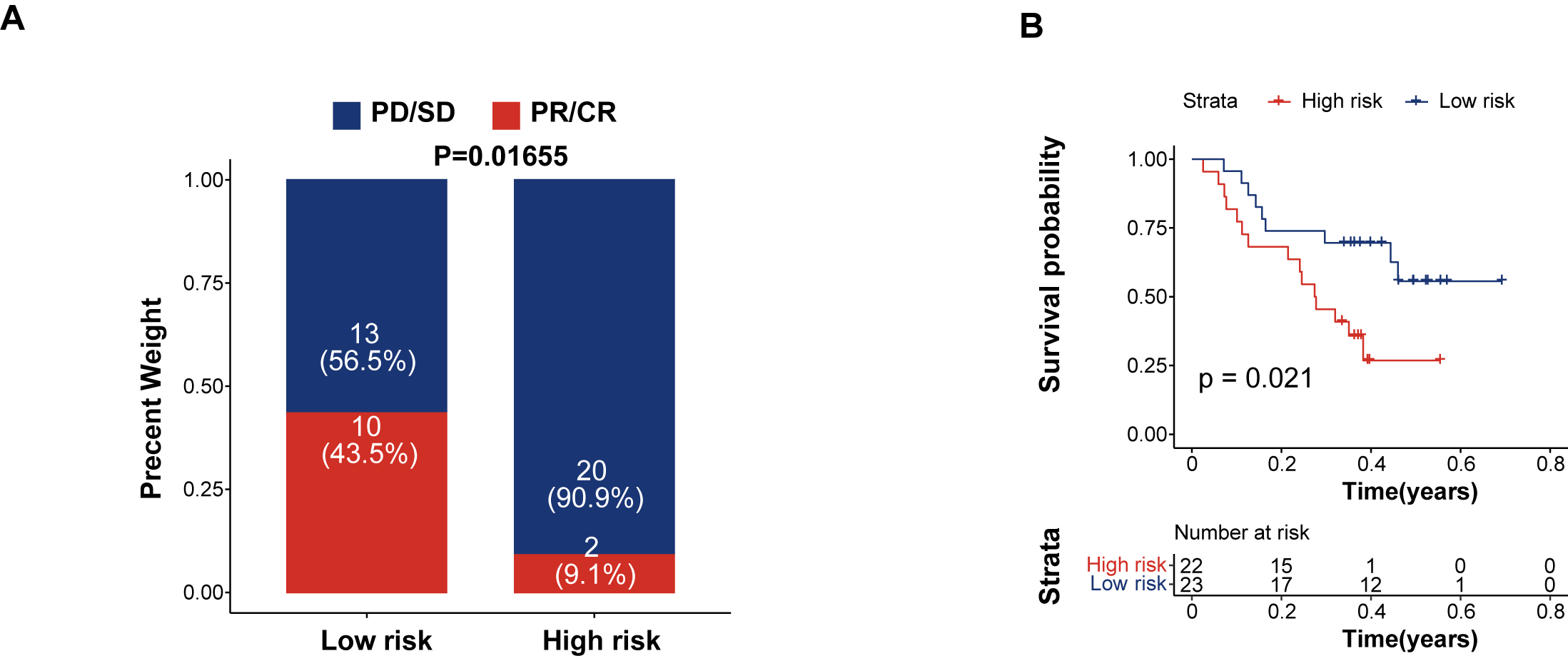

Supplement: Supplementary file 8 [file Image_7.tif]
